# Comparison of METS-IR and HOMA-IR for predicting new-onset CKD in middle-aged and older adults

**DOI:** 10.1186/s13098-023-01214-7

**Published:** 2023-11-14

**Authors:** Jihyun Yoon, Seok-Jae Heo, Jun-Hyuk Lee, Yu-Jin Kwon, Jung Eun Lee

**Affiliations:** 1 Department of Family Medicine, Anam Hospital, Korea University College of Medicine, 73 Goryeodae-ro, Seongbuk-gu, Seoul, 02481 Republic of Korea; 2 Division of Biostatistics, Department of Biomedical Systems Informatics, Yonsei University College of Medicine, Seoul, 03722 Republic of Korea; 3 Department of Family Medicine, Nowon Eulji Medical Center, Eulji University School of Medicine, Seoul, 01830 Republic of Korea; 4 Department of Medicine, Hanyang University Graduate School of Medicine, Seoul, 04763 Republic of Korea; 5 Department of Family Medicine, Yonsei University of College of Medicine, Yongin Severance Hospital, Yongin, 16995 Republic of Korea; 6 Division of Nephrology, Department of Internal Medicine, Yongin Severance Hospital, Yonsei University College of Medicine, Gyeonggi, Republic of Korea

**Keywords:** Chronic kidney disease, Insulin resistance, Metabolic score for insulin resistance

## Abstract

**Background:**

Chronic kidney disease (CKD) has emerged as a mounting public health issue worldwide; therefore, prompt identification and prevention are imperative in mitigating CKD-associated complications and mortality rate. We aimed to compare the predictive powers of the homeostatic model assessment for insulin resistance (HOMA-IR) and the metabolic score for insulin resistance (METS-IR) for CKD incidence in middle-aged and older adults.

**Methods:**

This study used longitudinal prospective cohort data from the Korean Genome and Epidemiology Study. A total of 10,030 participants, aged 40–69 years, residing in the Ansung or Ansan regions of the Republic of Korea, were recruited between 2001 and 2002 through a two-stage cluster sampling method. We compared the predictive powers of METS-IR and HOMA-IR for CKD prevalence and incidence, respectively. CKD prevalence was measured by the area under the receiver operating characteristic (ROC) curve (AUC), and the indices’ predictive performance for CKD incidence were assessed using Harrell’s concordance index and time-dependent ROC curve analysis.

**Results:**

A total of 9261 adults aged 40–69 years at baseline and 8243 adults without CKD were included in this study. The AUCs and 95% confidence intervals (CIs) of HOMA-IR and METS-IR for CKD prevalence at baseline were 0.577 (0.537–0.618) and 0.599 (0.560–0.637), respectively, with no significant difference (p = 0.337). The Heagerty’s integrated AUC for METS-IR in predicting CKD incidence was 0.772 (95% CI 0.750–0.799), which was significantly higher than that of HOMA-IR (0.767 [95% CI 0.742–0.791], p = 0.015).

**Conclusion:**

METS-IR surpassed HOMA-IR in predicting CKD incidence and was as effective as HOMA-IR in predicting CKD prevalence. This implies that METS-IR could be a valuable indicator for early detection and prevention of CKD among Korean adults.

**Supplementary Information:**

The online version contains supplementary material available at 10.1186/s13098-023-01214-7.

## Introduction

Chronic kidney disease (CKD) is a widespread and progressive condition that impacts more than 10% of the world's population, equating to over 800 million people globally [1]. CKD is characterized by the gradual loss of kidney function over time, which can lead to serious complications, such as cardiovascular disease (CVD), morbidity, and mortality, if left untreated [2, 3]. It is a major public health concern and requires early detection and proper management to prevent further kidney damage and improve patients' quality of life.

Several well-known conditions, such as type 2 diabetes mellitus (DM), hypertension (HTN), obesity, dyslipidemia, and metabolic syndrome, are recognized risk factors for the development of CKD [4–6]. These conditions are closely linked to insulin resistance (IR), which can affect the kidneys by promoting inflammation, oxidative stress, and endothelial dysfunction, contributing to the development and progression of CKD [7]. Previous studies have shown that IR in patients with CKD is closely linked to risk factors that contribute to CVD, such as chronic inflammation, endothelial dysfunction, and oxidative stress [8]. Therefore, early identification and management of IR can help prevent or delay the onset of CKD and its associated complications, highlighting the importance of routine monitoring and effective control of these conditions in high-risk individuals.

Despite the hyperinsulinemic-euglycemic clamp technique being the preferred method for measuring insulin sensitivity in humans, its invasiveness and impracticality makes it unsuitable for large-scale epidemiological studies [9]. Therefore, alternative non-insulin-based markers, such as the homeostasis model assessment for IR (HOMA-IR) [10] and the metabolic score for IR (METS-IR) [11], have been developed as substitutes for assessing IR. Some studies have demonstrated the effectiveness and usefulness of these two markers as surrogate indicators for CKD [12–16]. However, it is unclear which surrogate marker is more useful for predicting the prevalence and incidence of CKD. Therefore, this study aimed to compare the predictive power of METS-IR and HOMA-IR for the prevalence and incidence of CKD over a 14-year period in a substantial, community-based Korean prospective cohort.

## Methods

### Study population

**Fig. 1:**
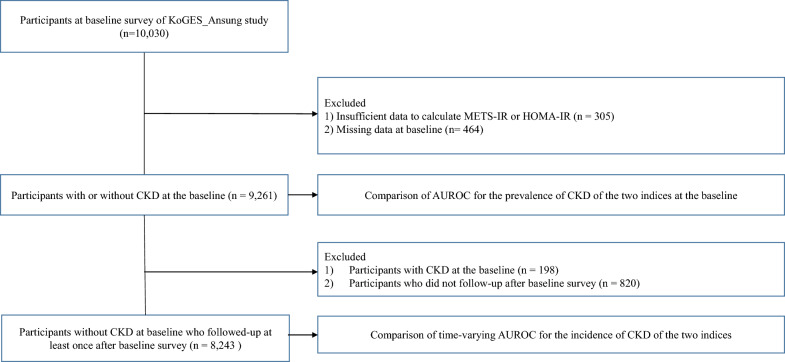
Flow chart of the study population. KoGES, Korean Genome and Epidemiology Study; *METS-IR* metabolic score for insulin resistance, *HOMA-IR* homeostatic assessment for insulin resistance, *CKD* chronic kidney disease, *AUROC* area under the receiver operating characteristic curve

We used data from a community-based cohort study (Korean Genome and Epidemiology Study, KoGES_Ansan_Ansung cohort). The KoGES_Ansan_Ansung cohort, which comprised adults aged 40–69 years, was conducted biennially from 2001–2002 (baseline) to the 7th follow-up in 2015–2016. From a total of 10,030 participants at baseline, we excluded participants with insufficient data to calculate METS-IR or HOMA-IR (n = 305) and those with missing data at baseline (n = 464). After exclusion, a total 9,261 participants (9,063 without CKD and 198 with CKD) were included in the first analysis to compare the discriminative power of METS-IR and HOMA-IR for detecting CKD prevalence. Subsequently, we excluded participants with CKD at baseline (n = 198) and those who did not follow-up data after baseline (n = 820). Finally, 8,243 participants were included in the analysis to compare the predictive performance of METS-IR and HOMA-IR for detecting CKD incidence. The flow chart of the study population is presented in the Fig. 1. All participants provided their written consent and agreed to participate in this study. The study was approved by the Institutional Review Board of Yongin Severance Hospital (IRB No. 9-2022-0090).

### Data collection

Anthropometric variables including the height (m), weight (kg), and waist circumference (cm) were measured by trained medical staff according to standard protocol. Body mass index (BMI) was calculated by weight (kg) divided by height (m) squared. After a 5-min rest, systolic blood pressure (SBP) and diastolic blood pressure (DBP) were measured twice, and the average of two of three measured values was recorded. Blood samples were collected after at least 8 h of fasting. Serum creatinine, total cholesterol (TC), high-density lipoprotein (HDL), triglyceride (TG), C-reactive protein (CRP), and glucose levels were enzymatically analyzed using a Chemistry Analyzer (Hitachi 7600, Tokyo, Japan by August 2002 and ADVIA 1650, Siemens, Tarrytown, NY from September 2002).

Smoking status was categorized into four groups: non-smoker, former smoker, occasional smoker, and daily smoker. Current smoker was further categorized into daily smokers and occasional smokers based on whether they smoked every day. Alcohol drinking status was categorized into non-drinker, former drinker, and current drinker. Physical activity was assessed using metabolic equivalent of task (MET)-hours per day (METs-h/day) using an International Physical Activity Questionnaire [17]. Total METs-h/day were calculated by multiplying the self-reported hours spent per day by the MET values calculated based on each activity type [18]. HTN was defined as having an SBP of 140 mmHg or more, a DBP of 90 mmHg or more, a diagnosis by a physician, or current treatment with antihypertensive medications [19]. DM was defined as having a fasting plasma glucose (FPG) level of 126 mg/dL or more; a plasma glucose level of 200 mg/dL or more 2 h after the 75 g oral glucose tolerance test; a glycosylated hemoglobin more than or equal to 6.5%; a diagnosis by a physician; or current treatment with anti-diabetic medication therapy [20]. Dyslipidemia was defined as a TC of 200 mg/dl or more, a diagnosis by a physician, or current treatment with anti-dyslipidemic medication therapy [21]. Obesity was defined as a BMI greater than 25 kg/m^2^ according to the 2018 Korean Society for the Study of Obesity guideline [22]. The detailed protocol used in KoGES is available on the website (https://nih.go.kr/ko/main/contents.do?menuNo=300583).

### Definitions of METS-IR and HOMA-IR

The METS-IR and HOMA-IR were calculated according to the following formulas: [11, 23]

1. $${\text{METS}} - {\text{IR = ln [2}} \times {\text{FPG}}\,\,{(}{{{\text{mg}}} \mathord{\left/ {\vphantom {{{\text{mg}}} {{\text{dL}}}}} \right. \kern-0pt} {{\text{dL}}}}) + {\text{fasting serum TG}}\,\,({{{\text{mg}}} \mathord{\left/ {\vphantom {{{\text{mg}}} {{\text{dL}}}}} \right. \kern-0pt} {{\text{dL}}}})] \times {\text{BMI}}\,\,{{{(}{{{\text{kg}}} \mathord{\left/ {\vphantom {{{\text{kg}}} {{\text{m}}^{{2}} }}} \right. \kern-0pt} {{\text{m}}^{{2}} }})} \mathord{\left/ {\vphantom {{{(}{{{\text{kg}}} \mathord{\left/ {\vphantom {{{\text{kg}}} {{\text{m}}^{{2}} }}} \right. \kern-0pt} {{\text{m}}^{{2}} }})} {{\text{ln}}}}} \right. \kern-0pt} {{\text{ln}}}}\,\,[{\text{HDL}} - {\text{C(}}{{{\text{mg}}} \mathord{\left/ {\vphantom {{{\text{mg}}} {{\text{dL}}}}} \right. \kern-0pt} {{\text{dL}}}})]$$
2. $${\text{HOMA - IR = [fasting serum insulin}}\,\,{(}{{{\mu U}} \mathord{\left/ {\vphantom {{{\mu U}} {{\text{mL}}}}} \right. \kern-0pt} {{\text{mL}}}}{)} \times {\text{FPG}}\,\,{{{(}{{{\text{mg}}} \mathord{\left/ {\vphantom {{{\text{mg}}} {{\text{dL}}}}} \right. \kern-0pt} {{\text{dL}}}})} \mathord{\left/ {\vphantom {{{(}{{{\text{mg}}} \mathord{\left/ {\vphantom {{{\text{mg}}} {{\text{dL}}}}} \right. \kern-0pt} {{\text{dL}}}})} {405}}} \right. \kern-0pt} {405}}]$$

### Definition of CKD

CKD was defined as an estimated glomerular filtration rate (eGFR) of less than 60 mL/min/1.73 m^2^. The eGFR was calculated using the CKD Epidemiology Collaboration (CKD-EPI) equation [24].

### Statistical analysis

All data are presented as means ± standard deviations (SDs) for continuous variables or numbers (percentages) for categorical variables. The independent two sample t-test was used to compare differences in continuous variables, including age, BMI, waist circumference (WC), SBP, DBP, FPG, TC, TG, HDL-C, CRP, eGFR, METS-IR, HOMA-IR, and METs, between participants without CKD and those with CKD or between participants who developed CKD or those who did not. The chi-squared test was used to compare differences in categorical variables, including smoking status, alcohol consumption, DM, HTN, and dyslipidemia, between two groups. For the 9261 participants at baseline, the receiver operating characteristics (ROC) was performed. The areas under the ROC curves (AUC) were used to compare the discriminative powers of METS-IR and HOMA-IR for CKD prevalence. Post hoc comparisons of the AUC of two indices were also performed. The cut-off points for such prediction were calculated by using the Youden index.

For the 8,243 participants without CKD at baseline, univariable and multivariable Cox proportional hazard regression analyses were performed to calculate the hazard ratio (HR) with a 95% confidence interval (CI) for the incidence of CKD according to single increments of METS-IR or HOMA-IR. In Model 1, we adjusted for age, sex, BMI, physical activity, smoking, and alcohol drinking. In Model 2, we adjusted for the Model 1 variables plus HTN, DM, dyslipidemia, and CRP.

The indices’ predictive performance for the incidence of CKD was assessed using Harrell’s concordance index and time-dependent ROC curve analyses. Heagerty’s integrated AUC (iAUC) and Heagerty’s AUC every 2 years were used as time-dependent AUCs with an age-adjusted survival analysis. Subgroup analyses by sex, presence of DM, presence of HTN, smoking status, and obesity status were performed, and the results are presented as a forest plot. All statistical analyses were conducted using SAS version 9.4 (SAS Institute Inc., Cary, NC) and R software (version 4.1.1; R Foundation for Statistical Computing, Vienna, Austria). Statistical significance was set at p-value < 0.05.

## Results

### Clinical characteristics of study participants

The baseline characteristics of the population with and without CKD at baseline survey are presented in Table 1. Among the 9261 participants, 198 participants had CKD at baseline survey. Participants with CKD were older (p < 0.001) and female (p = 0.014); they had higher BMIs (p = 0.003), WCs (p < 0.001), SBPs (p < 0.001), DBPs (p < 0.001), FPGs levels (p = 0.005), TC levels (p < 0.001), TG levels (p = 0.007), CRP levels (p = 0.039), HOMA-IR (p = 0.002), and METS-IR (p < 0.001). They were less likely to drink alcohol (< 0.001) and more likely to have DM (p = 0.010), HTN (p = 0.002), and dyslipidemia (p < 0.001).

**Table 1:** Baseline characteristics of the population with or without CKD at the baseline survey

Variable	Total	Without CKD	With CKD	p-value
N	9,261	9,063	198	
Age (years)	52.0 ± 8.9	51.8 ± 8.8	61.2 ± 7.8	< 0.001
Sex				0.014
Men	4426 (47.8%)	4349 (48.0%)	77 (38.9%)	
Women	4835 (52.2%)	4714 (52.0%)	121 (61.1%)	
Body mass index (kg/m^2^)	24.6 ± 3.2	24.6 ± 3.1	25.2 ± 3.1	0.003
Waist circumference (cm)	82.5 ± 8.8	82.4 ± 8.8	85.9 ± 9.1	< 0.001
SBP (mmHg)	121.3 ± 18.4	121.1 ± 18.4	129.8 ± 17.3	< 0.001
DBP (mmHg)	80.3 ± 11.5	80.2 ± 11.5	83.8 ± 10.3	< 0.001
FPG (mg/dL)	87.4 ± 21.5	87.3 ± 21.3	92.9 ± 27.9	0.005
Total cholesterol (mg/dL)	191.9 ± 35.7	191.5 ± 35.5	207.9 ± 40.9	< 0.001
Triglyceride (mg/dL)	161.2 ± 104.0	160.8 ± 104.0	180.3 ± 99.9	0.007
HDL-cholesterol (mg/dL)	44.7 ± 10.1	44.8 ± 10.1	43.3 ± 10.3	0.043
CRP	0.24 ± 0.54	0.24 ± 0.54	0.31 ± 0.47	0.039
eGFR (CKD-EPI)	92.0 ± 14.3	92.8 ± 13.2	53.4 ± 9.6	< 0.001
HOMA-IR	1.66 ± 1.15	1.66 ± 1.15	1.92 ± 1.17	0.002
METS-IR	37.87 ± 6.66	37.83 ± 6.66	39.81 ± 6.59	< 0.001
Smoking status^a^				0.098
Never smoker	5425 (58.6%)	5298 (58.5%)	127 (64.1%)	
Former smoker	1452 (15.7%)	1417 (15.6%)	35 (17.7%)	
Occasional smoker	268 (2.9%)	265 (2.9%)	3 (1.5%)	
Daily smoker	2116 (22.8%)	2083 (23.0%)	33 (16.7%)	
Alcohol drinking				< 0.001
Non-drinker	4239 (45.8%)	4126 (45.5%)	113 (57.1%)	
Former drinker	599 (6.5%)	577 (6.4%)	22 (11.1%)	
Current drinker	4423 (47.8%)	4360 (48.1%)	63 (31.8%)	
METs (hour/day)	23.65 ± 14.80	23.71 ± 14.81	21.10 ± 14.02	0.010
Diabetes mellitus	1127 (12.2%)	1088 (12.0%)	39 (19.7%)	0.002
Hypertension	2956 (31.9%)	2836 (31.3%)	120 (60.6%)	< 0.001
Dyslipidemia	3675 (39.7%)	3562 (39.3%)	113 (57.1%)	< 0.001

Values are presented as means ± standard deviations, medians (25th percentiles, 75th percentiles), or numbers (%)

*CKD* chronic kidney disease, *SBP* systolic blood pressure, *DBP* diastolic blood pressure, *FPG* fasting plasma glucose, *HDL* high-density lipoprotein, *AST* aspartate aminotransferase, *ALT* alanine aminotransferase, *CRP* C-reactive protein, *eGFR* estimated glomerular filtration rate, *METS*-*IR* metabolic score for insulin resistance, *HOMR-IR* homeostatic model assessment for insulin resistance, *MET* metabolic equivalent of task P-value for the comparison of baseline characteristics between participants with CKD and those without CKD at the baseline survey. Significance was set at a p < 0.05

^a^Never smokers comprised individuals who had never smoked or had smoked < 100 cigarettes in their lifetime. Former smokers comprised adults who had smoked ≥ 100 cigarettes in their lifetime and had quit smoking at the time of the survey. Current smoker was further categorized into daily smokers and occasional smokers based on whether they smoked every day

During the mean follow-up period of 10.9 years, among the 8,243 participants without CKD at baseline, a total of 1,506 (18.3%) later developed CKD. Table 2 shows baseline characteristics of the participants who developed CKD and those who did not during the follow-up period. Participants who developed CKD were older (p < 0.001) and female (p < 0.001); they had higher BMIs (p < 0.001), WCs (p < 0.001), SBPs (p < 0.001), DBPs (p < 0.001), FPG levels (p = 0.005), TC levels (p < 0.001), TG levels (p = 0.007), CRP levels (p = 0.039), HOMA-IR (p = 0.002), and METS-IR (p < 0.001). They were less likely to smoke (p < 0.001) and drink alcohol (p < 0.001), but were more likely to have type-2 DM (p < 0.001), HTN (p < 0.001), and dyslipidemia (p < 0.001).

**Table 2:** Baseline characteristics of the study population according to new-onset CKD

Variable	Total	No CKD	New-onset CKD	p-value
N	8243	6737	1506	
Age (years)	51.9 ± 8.8	50.3 ± 8.2	58.7 ± 7.8	< 0.001
Sex				< 0.001
Men	3964 (48.1%)	3371 (50.0%)	593 (39.4%)	
Women	4279 (51.9%)	3366 (50.0%)	913 (60.6%)	
Body mass index (kg/m^2^)	24.6 ± 3.1	24.5 ± 3.1	25.1 ± 3.3	< 0.001
Waist circumference (cm)	82.6 ± 8.8	82.0 ± 8.7	85.1 ± 8.7	< 0.001
SBP (mmHg)	121.1 ± 18.3	119.4 ± 17.4	128.6 ± 20.0	< 0.001
DBP (mmHg)	80.2 ± 11.4	79.6 ± 11.3	83.2 ± 11.6	< 0.001
FPG (mg/dL)	87.1 ± 20.6	86.4 ± 18.9	90.1 ± 26.5	< 0.001
Total cholesterol (mg/dL)	191.4 ± 35.0	190.1 ± 34.6	197.0 ± 36.4	< 0.001
Triglyceride (mg/dL)	160.8 ± 103.5	156.6 ± 100.8	179.4 ± 113.2	< 0.001
HDL-cholesterol (mg/dL)	44.7 ± 10.0	45.0 ± 10.0	43.5 ± 9.9	< 0.001
CRP	0.23 ± 0.54	0.22 ± 0.56	0.27 ± 0.43	< 0.001
eGFR (CKD-EPI)	92.9 ± 13.1	94.9 ± 12.5	84.3 ± 12.2	< 0.001
HOMA-IR	1.66 ± 1.15	1.62 ± 1.07	1.84 ± 1.47	< 0.001
METS-IR	37.85 ± 6.64	37.53 ± 6.53	39.28 ± 6.94	< 0.001
Current smoking (yes)†				< 0.001
1	4840 (58.7%)	3868 (57.4%)	972 (64.5%)	
2	1299 (15.8%)	1080 (16.0%)	219 (14.5%)	
3	244 (3.0%)	206 (3.1%)	38 (2.5%)	
4	1860 (22.6%)	1583 (23.5%)	277 (18.4%)	
Regular drinking				< 0.001
1	3750 (45.5%)	2901 (43.1%)	849 (56.4%)	
2	513 (6.2%)	404 (6.0%)	109 (7.2%)	
3	3980 (48.3%)	3432 (50.9%)	548 (36.4%)	
Mets (hour/day)	24.04 ± 14.90	23.89 ± 14.78	24.70 ± 15.42	0.064
Type 2 diabetes	969 (11.8%)	664 (9.9%)	305 (20.3%)	< 0.001
Hypertension	2579 (31.3%)	1858 (27.6%)	721 (47.9%)	< 0.001
Dyslipidemia	3217 (39.0%)	2526 (37.5%)	691 (45.9%)	< 0.001

Values are presented as means ± standard deviations, medians (25th percentiles, 75th percentiles), or numbers (%)

*CKD* chronic kidney disease, *SBP *systolic blood pressure, *DBP* diastolic blood pressure, *FPG* fasting plasma glucose, *HDL* high-density lipoprotein, *AST* aspartate aminotransferase, *ALT* alanine aminotransferase, *CRP C*-reactive protein, *eGFR* estimated glomerular filtration rate, *METS-IR* metabolic score for insulin resistance, *HOMR-IR* homeostatic model assessment for insulin resistance, *MET* metabolic equivalent of task P-value for the comparison of baseline characteristics between participants who developed CKD and those who did not develop CKD at the baseline survey. Significance was set at a p < 0.05

### Comparison of discriminative performance between METS-IR and HOMA-IR for prevalence of CKD

**Fig. 2:**
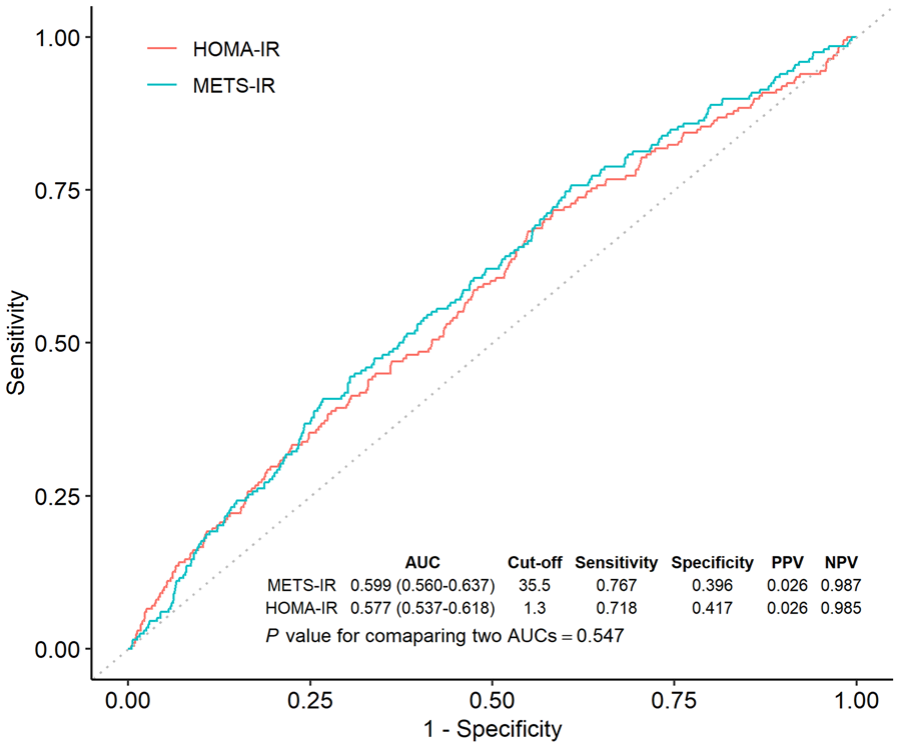
Comparison of predictive power for prevalent chronic kidney disease of metabolic score for insulin resistance and homeostasis model assessment for insulin resistance. A comparison was made between the predictive powers of METS-IR and HOMA-IR for prevalent CKD in the 9261 participants at baseline using the area under the receiver operating characteristic curves. The cut-off values for such prediction were determined using the Youden index. *ROC* receiver operating characteristic, *CKD* chronic kidney disease, *METS-IR* metabolic score for insulin resistance, *HOMA-IR* homeostatic assessment model for insulin resistance, *AUC* area under the receiver operating characteristic curve, *PPV* positive predictive value, *NPV* negative predictive value

The AUCs and 95% CIs of HOMA-IR and METS-IR for CKD prevalence at baseline were 0.577 (0.537–0.618) and 0.599 (0.560–0.637), respectively (Fig. 2). Although the AUC of METS-IR was higher than that of HOMA-IR, there was no significant difference (p = 0.337). The cut-off points of the discriminative performance of HOMA-IR and METS-IR for the prevalence of CKD were 1.3 and 35.5, respectively. The positive predictive value (PPV) for HOMA-IR was 0.026, with a negative predictive value (NPV) of 0.958. For METS-IR, the PPV was also 0.026, while the NPV was 0.987.

### Longitudinal relationship between METS-IR, HOMA-IR, and incidence of CKD

Table 3 shows the HR and 95% CI for CKD incidence according to single increments of METS-IR and HOMA-IR. The HR and 95% CI of CKD incidence for each 1-point increase in the METS-IR and the HOMA-IR were 1.04 and 1.03–1.04 (p < 0.001) and 1.12 and 1.08–1.15 (p < 0.001), respectively. There were significant associations between METS-IR, HOMA-IR, and CKD incidence even after adjusting for age, sex, BMI, physical activity, smoking, alcohol drinking, DM, HTN, dyslipidemia, and CRP.

**Table 3:** Cox proportional hazard regression model for CKD incidence of two different insulin resistance indices

	CKD Incidence		
	HR	95% CI	P-value
METS-IR (per 1 increment)			
Unadjusted	1.04	1.03–1.04	< 0.001
Model 1	1.06	1.05–1.08	< 0.001
Model 2	1.06	1.03–1.07	< 0.001
HOMA-IR (per 1 increment)			
Unadjusted	1.12	1.08–1.15	< 0.001
Model 1	1.06	1.03–1.10	< 0.001
Model 2	1.04	1.00–1.08	0.031

*METS-IR* metabolic score for insulin resistance, *HOMR*-*IR* homeostatic model assessment for insulin resistance, *CKD* chronic kidney disease, *HR* hazard ratio, *CI* confidence intervals, *BMI* body mass index, *HTN* hypertension, *DM* diabetes mellitus, *CRP* C-reactive protein

Model 1: Adjusted for age, sex, BMI, physical activity, smoking, and alcohol drinking

Model 2: Model 1, HTN, DM, dyslipidemia, and CRP

### Comparison of predictive performance of METS-IR and HOMA-IR for CKD incidence

Table 4 shows the age-adjusted Harrell’s C-index and Heagerty’s iAUC. The Harrell’s c-index of METS-IR was significantly higher than those of HOMA-IR (C-index: 0.772, 95% CI 0.760–0.784 for METS-IR vs 0.762, 0.750–0.774 for HOMA-IR, p < 0.001). The Heagerty’s iAUC of METS-IR was 0.775 (95% CI 0.750–0.799), which was significantly higher than that of HOMA-IR (Heagerty’s iAUC: 0.767, 95% CI 0.742–0.791) (p = 0.015). Heagerty’s incident/dynamic AUC for METS-IR was significantly higher than that of HOMA-IR up to 8 years, and there was no difference in Heagerty’s incident/dynamic AUC between the two indicators since then. The predictive performance of both METS-IR and HOMA-IR for CKD incidence was not significantly different from that of MET-IR alone.

**Table 4:** Comparison of the predictive power for CKD incidence between METS-IR and HOMA-IR using time-dependent receiver operating characteristics curves analysis

	Harrell’s C-index	Heagerty’s iAUC	Heagerty’s incident/dynamic AUC (2 years)	Heagerty’s incident/dynamic AUC (4 years)	Heagerty’s incident/dynamic AUC (6 years)	Heagerty’s incident/dynamic AUC (8 years)	Heagerty’s incident/dynamic AUC (10 years)	Heagerty’s incident/dynamic AUC (12 years)	Heagerty’s incident/dynamic AUC (14 years)
METS-IR, (1)	0.772 (0.760, 0.784)	0.775 (0.750, 0.799)	0.769 (0.742, 0.795)	0.793 (0.777, 0.810)	0.798 (0.780, 0.814)	0.780 (0.763, 0.797)	0.743 (0.721, 0.766)	0.745 (0.723, 0.767)	0.741 (0.707, 0.776)
HOMA-IR, (2)	0.762 (0.750, 0.774)	0.767 (0.742, 0.791)	0.740 (0.711, 0.766)	0.781 (0.763, 0.798)	0.788 (0.769, 0.805)	0.776 (0.757, 0.793)	0.743 (0.719, 0.766)	0.740 (0.717, 0.764)	0.737 (0.704, 0.771)
METS-IR + HOMA-IR, (3)	0.773 (0.761, 0.784)	0.776 (0.752, 0.800)	0.768 (0.739, 0.795)	0.794 (0.778, 0.810)	0.798 (0.780, 0.814)	0.780 (0.763, 0.798)	0.744 (0.721, 0.766)	0.746 (0.724, 0.768)	0.742 (0.708, 0.776)
Difference (1)–(2)	0.010 (0.006, 0.014)	0.008 (0.001, 0.016)	0.029 (0.019, 0.041)	0.013 (0.007, 0.019)	0.010 (0.004, 0.016)	0.004 (0.001, 0.010)	− 0.000 (− 0.007, 0.007)	0.005 (− 0.003, 0.013)	0.003 (− 0.008, 0.014)
Difference (1)–(3)	− 0.000 (− 0.001, 0.000)	− 0.001 (− 0.003, 0.000)	0.002 (− 0.000, 0.004)	− 0.001 (− 0.003, 0.001)	− 0.000 (− 0.002, 0.001)	− 0.000 (− 0.002, 0.001)	− 0.001 (− 0.003, 0.001)	− 0.001 (− 0.004, 0.001)	− 0.001 (− 0.004, 0.001)
Difference (2)–(3)	− 0.010 (− 0.014, − 0.007)	− 0.009 (− 0.018, − 0.002)	− 0.028 (− 0.039, − 0.018)	− 0.014 (− 0.020, − 0.008)	− 0.010 (− 0.017, − 0.005)	− 0.005 (− 0.010, 0.000)	− 0.000 (− 0.007, 0.005)	− 0.006 (− 0.014, 0.002)	− 0.004 (− 0.015, 0.007)
P-value: (1) vs. (2)	< 0.001	0.015	< 0.001	< 0.001	< 0.001	0.035	0.532	0.135	0.314
P-value: (1) vs. (3)	0.507	0.112	0.973	0.078	0.190	0.367	0.249	0.097	0.152
P-value: (2) vs. (3)	< 0.001	0.003	< 0.001	< 0.001	< 0.001	0.047	0.444	0.104	0.245

*METS-IR* metabolic score for insulin resistance, *HOMA-IR* homeostatic model assessment for insulin resistance, *iAUC* integrated area under the receiver operating characteristic curve, *AUC* area under the receiver operating characteristic curve. All values were adjusted for age

**Fig. 3:**
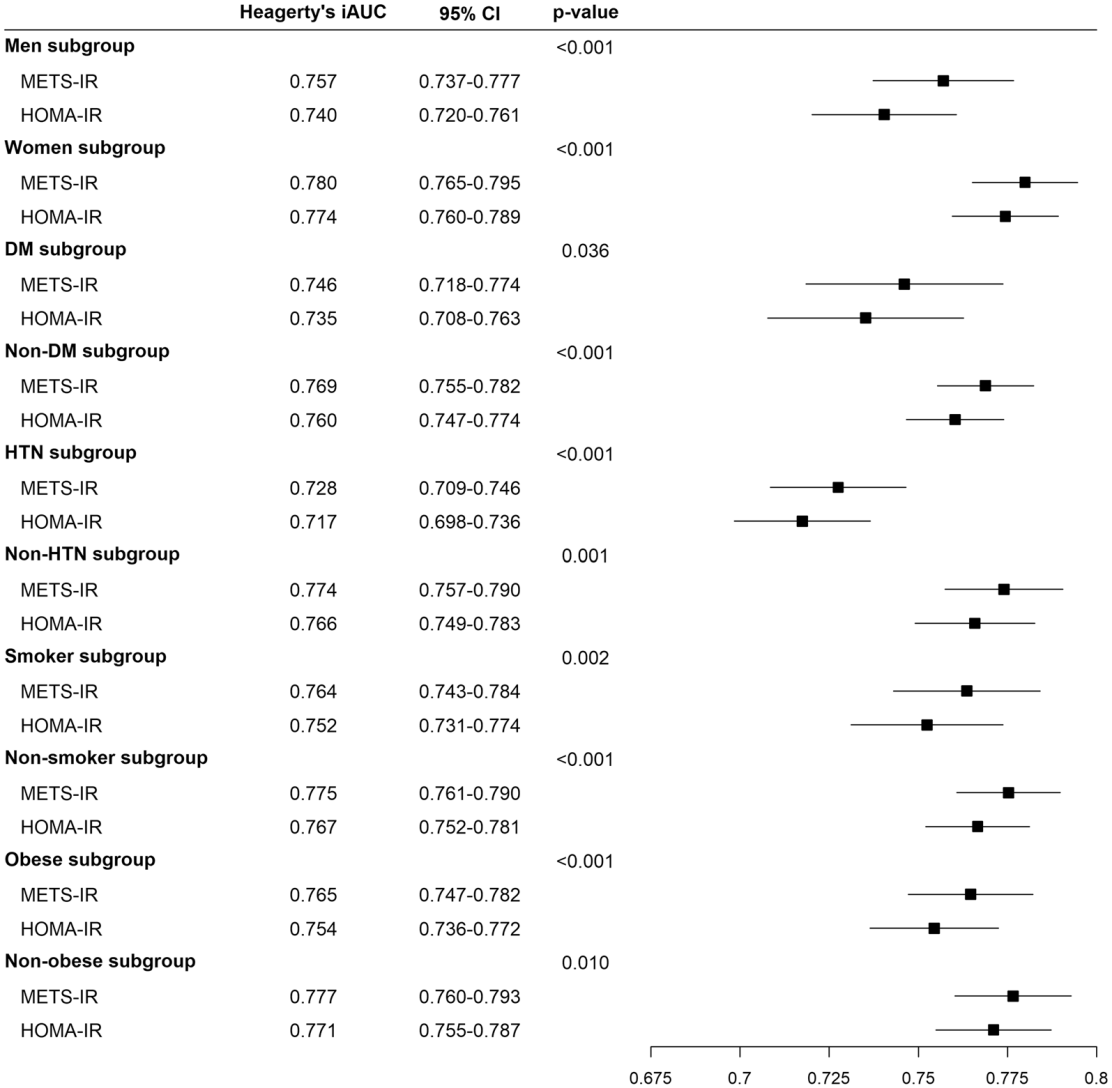
Forest plot showing the predictive power for incident chronic kidney disease by subgroups according to sex and diabetes mellitus, hypertension, smoking, and obesity status. During the 14 year follow-up period, Heagerty’s integrated AUC was used as time-dependent AUC with an unadjusted survival analysis framework approach. A bootstrapping method to calculate the differences and 95% CI of Heagerty’s integrated AUC between the METS-IR and HOMA-IR. *DM* diabetes mellitus, *HTN* hypertension, *METS-IR* metabolic score for insulin resistance, *HOMA-IR* homeostatic model assessment for insulin resistance, *AUC* area under the receiver operating characteristic curve, *CI* confidence interval

Figure 3 shows the predictive performance for CKD incidence by subgroup analysis according to sex, presence of DM, presence of HTN, smoking status, and obesity status. The predictive performances of METS-IR were significantly higher those of HOMA-IR regardless of sex (men; p < 0.001 and women; p < 0.001), presence of DM (DM; p = 0.036 and non-DM; p < 0.001), presence of HTN (HTN; p < 0.001 and non-HTN; p = 0.001), smoking status (smoker; p = 0.002 and non-smoker; p < 0.001), and obesity status (obese; p < 0.001 and non-obese; p = 0.010).

## Discussion

We found significant associations between METS-IR and incidence of CKD, as well as between HOMA-IR and incidence of CKD using the data from a large community-based prospective cohort with a 14-year follow-up. Our results suggest that there was no significant difference in the predictive abilities of METS-IR and HOMA-IR for CKD prevalence at baseline. Considering the comparable predictive abilities of METS-IR and HOMA-IR for CKD prevalence, our findings suggest that it may be more practical to use lower cut-off values of 1.3 for HOMA-IR and 35.5 for METS-IR in clinical settings, rather than the widely accepted cut-off value of 2.5 for defining IR.

Our results showed that, contrastingly, METS-IR had a higher predictive ability for CKD incidence compared to HOMA-IR (Heagerty's iAUC: 0.775 vs. 0.767). Even after the subgroup analysis of potential risk factors for CKD incidence, METS-IR remained a better predictor. Furthermore, the combination of both indices did not significantly enhance the predictive power of CKD incidence compared with using METS-IR alone. 

There could be several reasons for the superior predictive ability of METS-IR over HOMA-IR for CKD incidence. One possible explanation is that METS-IR is better at describing peripheral IR compared to HOMA-IR [11]. CKD could be more closely associated with peripheral IR than hepatic IR. This is because post-receptor signal defects, such as reduced PI3K/Akt activity in skeletal muscle and adipose tissue, are recognized as the primary cause of insulin resistance in patients with CKD [7, 25]. Previous studies have demonstrated that dysfunction in the PI3K/Akt signaling pathway may lead to the inhibition of anti-lipolytic function, a rise in free fatty acid release, elevation in serum TG levels, and the onset of ectopic fat deposition, ultimately leading to lipotoxicity and IR [26].

Second, the superiority of METS-IR is its effectiveness as a screening and predictive tool for diseases related to metabolic syndrome [27, 28]. Metabolic syndrome is closely linked to CKD, as the kidney is a highly sensitive target organ for metabolic syndrome [29]. Numerous studies have provided significant evidence linking IR and chronic inflammation to metabolic syndrome, which can lead to anomalies in lipid and glucose metabolism [30–32].

Third, METS-IR includes BMI, which can provide valuable insights into the nutritional status of individuals with CKD. Given that many patients with CKD are affected by protein-energy malnutrition, they may present with a micro-inflammatory state, low BMI, progressive skeletal muscle wasting, and insufficient nutritional and caloric intake [33]. Studies have indicated that individuals with CKD frequently suffer from anorexia, leading to a decrease in daily food intake, which can result in malnutrition and reduced plasma albumin levels, negatively impacting muscle protein synthesis and metabolism [34]. Therefore, METS-IR is deemed to be a more reliable predictor of CKD than HOMA-IR, since it consists of three direct components of metabolic syndrome (glucose, TG, and HDL-C levels) and one indirect component (BMI). A study conducted among the Chinese population demonstrated that METS-IR is linked to CKD and albuminuria. For each 1-unit increase in METS-IR, the risk of both CKD and albuminuria rose by 2%. It has also been reported that the higher the METS-IR, the higher the risk of CKD (odds ratio (OR): 1.02, 95% CI 1.01–1.03) [35].

Fourth, METS-IR reflects the impact of chronic inflammation on IR in individuals with CKD. In the early stages of CKD, patients typically have elevated levels of circulating inflammatory cytokines, such as tissue necrosis factor alpha (TNF-α), interleukin-6, interferon gamma, and lipopolysaccharide. These circulating inflammatory cytokines are produced by various organs in the body, including the kidneys, adipocytes, liver, or muscles [36, 37]. The activation of p44/42 kinase by TNF-α suppresses early insulin receptor signaling, which disrupts insulin's antilipolytic function and triggers the production of free fatty acids through lipolysis in adipose tissues, leading to increased serum TG levels [38]. Additionally, HDL-C suppresses the production of several pro-inflammatory cytokines and chemokines, and reduces the expression of adhesion molecules, demonstrating its anti-inflammatory properties [39]. Therefore, METS-IR is considered to be a better indicator of chronic inflammation than HOMA-IR.

The final possible explanation for the superior predictive power of METS-IR over HOMA-IR is that Koreans typically have a smaller pancreatic volume and higher pancreatic fat content than their Western counterparts with similar BMIs and body fat indices; this can result in reduced pancreatic secretions. Therefore, using HOMA-IR may underestimate IR in Koreans [40].

Our study revealed an interesting finding that the predictive power of both METS-IR and HOMA-IR for CKD incidence increased until year 8 of follow-up, but decreased after year 10, leading to a similar predictive power. This suggests that factors other than IR may affect CKD development over time. One possible factor is the function of pancreatic beta cells, which have been shown to be impaired without a change in pancreatic beta cell mass in patients with CKD. Furthermore, beta cell dysfunction alone may be enough to cause glucose intolerance [41]. Moreover, Koreans are known to have a genetic trait that lowers their insulin secretion compared to their Western counterparts [42]. Therefore, in the presence of IR, beta cells typically increase insulin secretion several-fold as a compensatory response [43]. However, Koreans are often unable to increase pancreatic insulin secretion sufficiently, regardless of obesity [44]. Second, the causes of IR in CKD are complex and can be influenced by multiple factors. Various risk factors, such as physical inactivity, oxidative stress, vitamin D deficiency, metabolic acidosis, anemia, and microbial toxins, may contribute to IR in CKD. Additionally, the impact of these factors on patients with CKD may vary over time. [4] For example, a study has shown that in patients with CKD, vitamin D deficiency can inhibit the secretion of insulin in response to glucose stimulation. Additionally, the same study found that vitamin D supplementation can raise insulin levels in vivo [45]. Furthermore, several risk factors, such as autoimmune diseases, genetic disorders, environmental pollution, and increased prevalence of DM and HTN, have been identified as important contributors to CKD [46–49]. Further studies are required to identify the precise molecular mechanisms responsible for the pathogenesis of IR in CKD and investigate other high-risk factors to predict CKD.

HOMA-IR has been validated and is a useful surrogate index to measure IR in clinical application; however, the serum insulin is not a routine laboratory measurement in usual clinical settings. Therefore, potential alternative indicators of IR, such as triglyceride-glucose (TyG) index, have also been studied and validated. In the present study, the AUC for HOMA-IR and TyG were not significantly different at baseline (p for AUC comparison = 0.693), and the AUC for HOMA-IR and TyG were also not significantly different at baseline (p for AUC comparison = 0.382) (Additional file 1: Figure S1). Interestingly, Harrell’s C index for TyG was significantly greater than that of HOMA-IR, but there was no significant difference in Harrell's C index between METS-IR and TyG. Additionally, Heagerty's iAUC for TyG was significantly higher than that of HOMA-IR (p = 0.007); however, Heagerty's iAUC for METS-IR was significantly higher than that of TyG (p = 0.011) (Additional file 1: Tables S1, S2).

This study had some limitations. Firstly, the study population was limited to Koreans; therefore, the findings may not be applicable to other ethnic groups. Second, since we used metabolic parameters and anthropometric measurements which were measured in a baseline survey, changes in METS-IR and HOMA-IR during the follow-up period could not be considered. Despite these weaknesses, to the best of our knowledge, this study is the first to investigate the effect of METS-IR and HOMA-IR on CKD incidence using large-scale population-based data.

In the present study, we found that both METS-IR and HOMA-IR have a high predictive power for CKD development, but METS-IR was superior to HOMA-IR. Given its convenience and economic feasibility compared to HOMA-IR, METS-IR could be an effective tool for early detection and prevention of CKD in the Korean population.

### Supplementary Information

**Additional file 1: Figure S1. **Comparison of predictive power for prevalent chronic kidney disease of metabolic score for insulin resistance, homeostasis model assessment, and triglyceride –glucose index for insulin resistance. A comparison was made between the predictive powers of METS-IR and HOMA-IR for prevalent CKD in the 9261 participants at baseline using area under the receiver operating characteristic curves. The cut-off values for such prediction were determined using the Youden index. ROC receiver operating characteristic, CKD chronic kidney disease, METS-IR metabolic score for insulin resistance, HOMA-IR homeostatic assessment model for insulin resistance, TyG triglyceride-glucose index, AUC area under the receiver operating characteristic curve, PPV positive predictive value, NPV negative predictive value. **Table S1.** Comparison of the predictive power for CKD incidence between HOMA-IR and TyG using time-dependent receiver operating characteristics curves analysis. **Table S2.** Comparison of the predictive power for CKD incidence between METS-IR and TyG using time-dependent receiver operating characteristics curves analysis.

## Data Availability

The dataset used in this study can be provided after a Korea Centers for Disease Control and Prevention review and evaluation of the research plan (http://www.cdc.go.kr/CDC/eng/main.jsp).
