# Supplementary material for: Comparison of METS-IR and HOMA-IR for predicting new-onset CKD in middle-aged and older adults
Source: Diabetol Metab Syndr. 2023 Nov 14;15:230. doi: 10.1186/s13098-023-01214-7 (PMC10644442; doi:10.1186/s13098-023-01214-7)
Supplement: Supplementary file 1 — Additional file 1: Figure S1. Comparison of predictive power for prevalent chronic kidney disease of metabolic score for insulin resistance, homeostasis model assessment, and triglyceride –glucose index for insulin resistance. A comparison was made between the predictive powers of METS-IR and HOMA-IR for prevalent CKD in the 9261 participants at baseline using area under the receiver operating characteristic curves. The cut-off values for such prediction were determined using the Youden index. ROC receiver operating characteristic, CKD chronic kidney disease, METS-IR metabolic score for insulin resistance, HOMA-IR homeostatic assessment model for insulin resistance, TyG triglyceride-glucose index, AUC area under the receiver operating characteristic curve, PPV positive predictive value, NPV negative predictive value. Table S1. Comparison of the predictive power for CKD incidence between HOMA-IR and TyG using time-dependent receiver operating characteristics curves analysis. Table S2. Comparison of the predictive power for CKD incidence between METS-IR and TyG using time-dependent receiver operating characteristics curves analysis. [file 13098_2023_1214_MOESM1_ESM.docx]

**Additional file materials**

**Comparison of METS-IR and HOMA-IR for predicting new-onset CKD in middle- aged and older adults**

Jihyun Yoon^1†^, Seok-Jae Heo^2†^, Jun-Hyuk Lee^3,4^, Yu-Jin Kwon^5*^, Jung Eun Lee^6*^

^1^Department of Family Medicine, Korean University Anam Hospital, 73 Goryeodae-ro, Seongbuk-gu, Seoul 02481, Republic of Korea

^2^Division of Biostatistics, Department of Biomedical Systems Informatics, Yonsei University College of Medicine, Seoul 03722, Republic of Korea

^3^Department of Family Medicine, Nowon Eulji Medical Center, Eulji University School of Medicine, Seoul 01830, Republic of Korea

^4^Department of Medicine, Hanyang University Graduate School of Medicine, Seoul 04763, Republic of Korea

^5^ Department of Family Medicine, Yonsei University of College of Medicine, Seoul, 03722, Republic of Korea

^6^ Division of Nephrology, Department of Internal Medicine, Yongin Severance Hospital, Yonsei University College of Medicine, Gyeonggi, Republic of Korea

^†^These authors contributed equally to this work and share first authorship.

* These authors contributed equally to this work.

*** Corresponding authors:**

Jung Eun Lee, MD, PhD

Clinical Professor, Division of Nephrology, Department of Internal Medicine, Yongin Severance Hospital, Yonsei University College of Medicine, Seoul 03722, Republic of Korea
Tel: +82-31-5189-8757, Mobile: +82-10-8730-2123,E-mail: [sw0615@yuhs.ac](mailto:sw0615@yuhs.ac)

Yu-Jin Kwon MD, PhD

Clinical Assistant Professor

Department of Family Medicine, Yongin Severance Hospital, Yonsei University College of Medicine, Seoul 03722, Republic of Korea Tel: +82-31-5189-8777,Mobile: +82 10-4180-7414
Fax: +82-31-5189-8567,Email: [digda3@yuhs.ac](mailto:digda3@yuhs.ac)


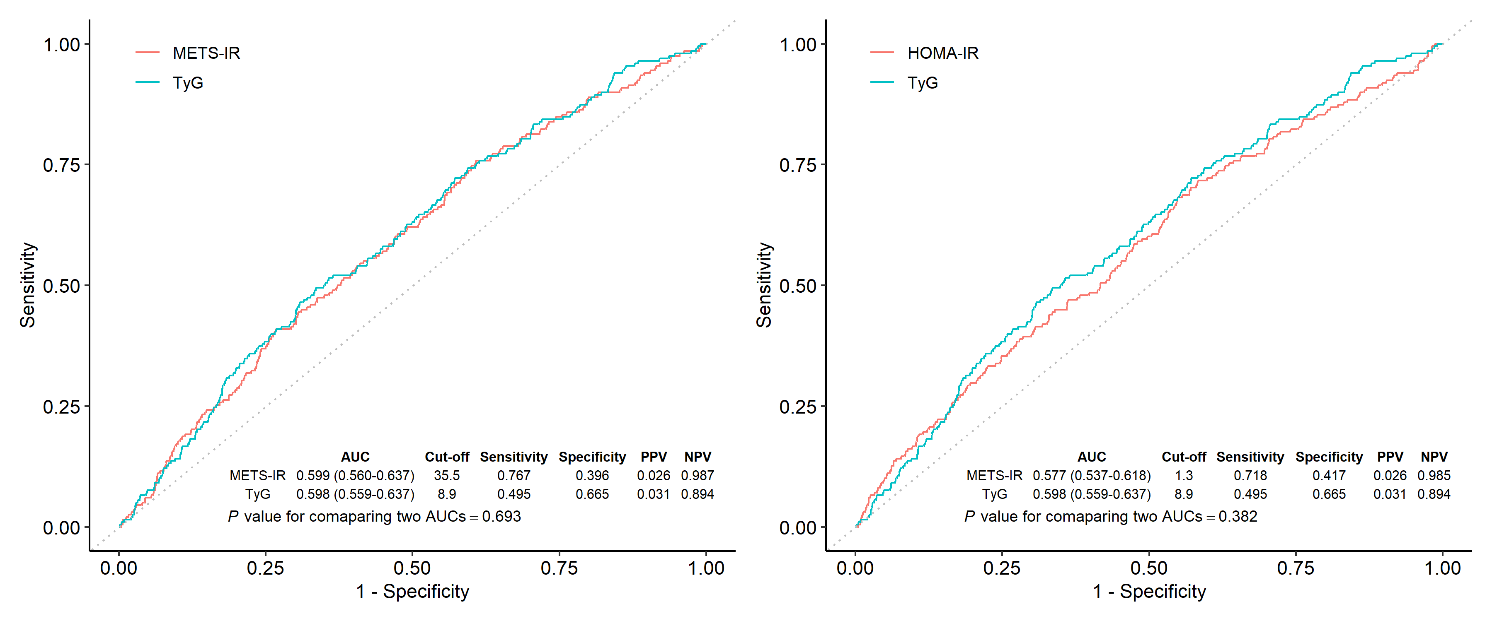


**Additional file 1: Figure 1.** Comparison of predictive power for prevalent chronic kidney disease of metabolic score for insulin resistance, homeostasis model assessment, and triglyceride –glucose index for insulin resistance. A comparison was made between the predictive powers of METS-IR and HOMA-IR for prevalent CKD in the 9,261 participants at baseline using area under the receiver operating characteristic curves. The cut-off values for such prediction were determined using the Youden index. ROC, receiver operating characteristic; CKD, chronic kidney disease; METS-IR, metabolic score for insulin resistance; HOMA-IR, homeostatic assessment model for insulin resistance; TyG, triglyceride-glucose index; AUC, area under the receiver operating characteristic curve; PPV, positive predictive value; NPV, negative predictive value

**Additional file 1: Table 1. Comparison of the predictive power for CKD incidence between HOMA-IR and TyG using time-dependent receiver operating characteristics curves analysis**

|  | **Harrell's C index** | **Heagerty's iAUC** |
| --- | --- | --- |
| HOMA-IR, (1) | 0.762 (0.750, 0.774) | 0.767 (0.741, 0.790) |
| TyG, (2) | 0.770 (0.758, 0.781) | 0.775 (0.750, 0.799) |
| HOMA-IR+TyG, (3) | 0.770 (0.758, 0.782) | 0.777 (0.751, 0.798) |
| Difference (1)-(2) | -0.007 (-0.011, -0.004) | -0.008 (-0.016, -0.001) |
| Difference (1)-(3) | -0.008 (-0.011, -0.004) | -0.009 (-0.017, -0.003) |
| Difference (2)-(3) | -0.000 (-0.001, 0.000) | -0.001 (-0.003, 0.000) |
| P-value: (1) vs. (2) | <0.001 | 0.007 |
| P-value: (1) vs. (3) | <0.001 | 0.002 |
| P-value: (2) vs. (3) | 0.234 | 0.082 |

age-adjusted Harrell’s c-index and Heagerty’s iAUC.

**Additional file 1: Table 2. Comparison of the predictive power for CKD incidence between METS-IR and TyG using time-dependent receiver operating characteristics curves analysis**

|  | **Harrell's C index** | **Heagerty's iAUC** |
| --- | --- | --- |
| METS-IR, (1) | 0.772 (0.760, 0.784) | 0.775 (0.750, 0.799) |
| TyG, (2) | 0.770 (0.758, 0.781) | 0.766 (0.741, 0.789) |
| METS-IR+TyG, (3) | 0.774 (0.762, 0.786) | 0.776 (0.750, 0.799) |
| Difference (1)-(2) | 0.003 (-0.000, 0.006) | 0.008 (0.001, 0.016) |
| Difference (1)-(3) | -0.001 (-0.003, 0.000) | -0.001 (-0.003, 0.001) |
| Difference (2)-(3) | -0.004 (-0.006, -0.002) | -0.009 (-0.017, -0.003) |
| P-value: (1) vs. (2) | 0.091 | 0.011 |
| P-value: (1) vs. (3) | 0.064 | 0.109 |
| P-value: (2) vs. (3) | <0.001 | 0.004 |

age-adjusted Harrell’s c-index and Heagerty’s iAUC.
